# General Practitioners' recommendations of self-directed-exercises for musculoskeletal problems and perceived barriers and facilitators to doing so: a mixed methods study

**DOI:** 10.1186/s12913-018-3799-x

**Published:** 2018-12-27

**Authors:** Toby Gillman, Kelly Ann Schmidtke, Victoria Manning, Ivo Vlaev

**Affiliations:** 1 Centre for Health Policy, Imperial College London, London, UK; 2 Warwick Business School, University of Warwick, Coventry, UK; 3 MSk Lab, Department of Surgery and Cancer, Faculty of Medicine, Imperial College London, London, UK

**Keywords:** Implementation, Guidelines, Musculoskeletal disease, Primary care, Behavioral science

## Abstract

**Background:**

Musculoskeletal problems substantially impact the demand for and the finances of the United Kingdom’s National Health Service. Some of this demand and cost could be alleviated if patients use self-directed-exercises. The present study aims first to establish whether general practitioners already recommend self-directed-exercises and second to describe barriers and facilitators to making such recommendations.

**Method:**

The design of the current study included surveys and interviews. The surveys were designed to draw out participants’ tendency to recommend self-directed-exercises and their behavioral drivers to do so. The drivers investigated include 14 domains described by the Theoretical Domains Framework. The surveys were completed online and the responses were analyzed using descriptive reports and regression analyses. The interviews were designed to more fully understand participants’ experiences recommending self-directed-exercises according to the same framework. The interviews were audio-taped, transcribed, and thematically analyzed.

**Results:**

The survey found that the following domains significantly predicted participants’ tendency to recommend self-directed-exercises: Environmental contexts and resources, Goals, Intentions, Knowledge, Memory attention and decision processes, and Social/professional role. The interviews brought out four themes that could be leveraged to increase general practitioners’ tendency to recommend self-directed-exercises: (1) Practitioners’ beliefs about self-directed-exercises being effective, (2) Patients’ motivations to engage in self-directed-exercises, (3) Time constraints, and (4) The ease with which practitioners can recommend self-directed-exercises.

**Conclusions:**

Most general practitioners already recommend self-directed-exercises, though they note significant barriers that may prevent them from doing so. General practitioners’ tendency to recommend self-directed-exercises would be bolstered by creating a respected central resource of exercise pamphlets. These pamphlets should clearly describe how different self-directed-exercises should be performed and evidence supporting their effectiveness.

**Electronic supplementary material:**

The online version of this article (10.1186/s12913-018-3799-x) contains supplementary material, which is available to authorized users.

## Background

Musculoskeletal conditions are characterized by damage or disorder to joints or other tissues causing pain or discomfort [1]. Musculoskeletal problems substantially impact the demand for and the finances of the United Kingdom’s National Health Service (NHS). Regarding demand, musculoskeletal problems bring approximately 100,000 people to general practices every day [2], accounting for about one-third of consultations [3]. Regarding finances, the NHS spends approximately £5.4 billion on musculoskeletal problems annually, making them the fourth largest disease group in terms of spending [4]. Some of this demand and cost could be alleviated if general practitioners recommended that patients use self-directed-exercises. The present study aims first to establish whether general practitioners already recommend self-directed-exercises and second to describe the barriers and facilitators general practitioners experience to making such recommendations.

Self-directed-exercises can improve the wellbeing of patients affected by musculoskeletal problems and many patients are happy to use them [5, 6]. For example, eccentric exercises (lengthening muscles under strain) benefit patients affected by tennis elbow more than conservative management after 6 weeks and more than corticosteroid injections after 52 weeks [7–10]. A number of other exercises for tennis elbow exist along with online resources to help patients use them [11–14]. There is notable demand for these online resources. For example a YouTube video demonstrating the “Taylor Twist” exercise for tennis elbow (twisting the wrists as a bar is shifted from a horizontal to vertical position) has over 400,000 views as of June 2018 [15]. A 2015 Cochrane review suggests that the beneficial effects of exercises extend to knee osteoarthritis, and four studies in this review looked specifically at self-directed-exercises [16–19]. Among the four studies was O’Reilly et al.’s that sought to help new patients take up self-directed-exercises. Surprisingly, O’Reily et al.’s study found that 24 patients were preforming self-directed-exercises before they were contacted by the research team [16]. This finding emphasizes that many patients are already motivated to take up self-directed-exercises.

Clinical guidelines advocate that general practitioners recommend self-directed-exercises to patients suffering from musculoskeletal problems, but such guidelines are often not clear as to when recommendations should be issued and what they should contain. For example, the National Institute for Health and Care Excellence (NICE) guidelines identify “strengthening exercise and aerobic fitness training” as central to the treatment of osteoarthritis. This advice is echoed by the European League Against Rheumatism [20]. The NICE guidelines also recommend a “structured exercise program” for the early treatment of non-specific lower back pain [21]. NICE states that self-directed-exercises can reduce patients’ pain and disability [22] in a cost-effective manner [21]. In addition, self-directed-exercises fit well in the NHS Five-Year Forward View “to support people to manage their own health” [23]. Fear that self-directed-exercises are harmful is largely unwarranted. The Cochrane reviews on exercise in knee [24] and hip [25] osteoarthritis and meta-analyses of exercises for tendinopathy [26] and back pain [27] find no significantly harmful outcomes.

While the above information demonstrates patient demand and organizational support for self-directed-exercises, it is unknown whether general practitioners routinely recommend self-directed-exercises to patients affected by musculoskeletal problems. Cottrell et al.’s review of general practitioners’ attitudes, beliefs, and behaviors regarding exercise for chronic knee pain concluded that self-directed-exercises are under-recommended [28]. Other evidence suggests that recommending self-directed-exercises may be common practice among some general practitioners, as some already undertake specific training in musculoskeletal medicine [29].

The current study has two aims (i.e., objectives). Aim one is to describe whether general practitioners recommend self-directed-exercises to patients affected by musculoskeletal problems. Aim two is to describe the barriers and facilitators general practitioners experience to making such recommendations.

## Methods

Before the current study was conducted the local research and development department determined that the current study did not need ethical approval and could continue as a quality improvement service evaluation conducted with the university. The design of the current study included surveys and follow-up interviews, as recommended by the Medical Research Council’s mixed-method approach [30]. Below, the survey method and interview method are described.

### Survey method

#### Survey recruitment

The study aimed to recruit at least 100 practitioners form England. As this was exploratory research, no a priori sample size calculations were performed. Initially Aylesbury Vale Clinical Commissioning Group invited practitioners who routinely saw patients presenting with musculoskeletal problems to participate. These potential participants were sent an anonymous online survey via SurveyMonkey®. To increase the participant pool, these participants were invited to send the survey to other practitioners who routinely saw patients presenting with musculoskeletal problems. Then to further increase the participant pool, Imperial College Primary Care Research Network sent the survey to its practitioners, and The Arthritis UK Lead for Primary Care and Primary Care Rheumatology Society sent the survey to its members. As it is unknown how many practitioners were invited to take up the survey, a participation rate could not be calculated.

The survey was completed by 117 participants. All participants were from England. Most participants were from the South Eastern region (*N* = 68). Fewer participants were from the Northern region (*N* = 19), and the Midlands and Eastern regions (*N* = 11). Fewer than 10 participants were from any other single region, 4 participants did not disclose their location. Regarding participants’ roles in primary care, 108 were general practitioners, 8 had other positions, e.g., physiotherapy, and 1 did not say. Moving forward the survey results will focus on the 108 participants who identified as general practitioners. Of these 108 participants 61 identified as female and 47 identified as male. Of the 108 participants, 105 revealed their age, and the mean was 42.81 (*Mdn* = 44, *SD* = 12.73). Of the 108 participants 93 revealed their years of experience in primary care, and the mean was 14 years (*Mdn* = 14, *SD* = 9.39). The participants’ characteristics are described in Table 1. Note that the present sample of general practitioners is only a small proportion of the total number of general practitioners in England (41,985 in 2016) but a fair representation of general practitioners’ gender (52.1% in 2016) and age (most general practitioners were between 35 and 44 years old in 2016) [31].

**Table 1:** Participant Characteristics

Participants Characteristics	Descriptive Statistics (e.g., Mdn)
Number	*N* = 108
Age	Mdn = 44, SD = 12.73
Female	*N* = 61
Male	*N* = 47
Years of Experience in Primary Care	Mdn = 14, *SD* = 9.39

#### Survey Materials

The content of the survey is briefly described here. A full copy is provided in Additional file 1. The survey’s items were discussed with a convenient sample of three general practitioners and academic experts in primary care and service delivery with specific interests in musculoskeletal care. These experts confirmed that the items were relevant and worded appropriately. No additional items or changes were suggested. As this is a new survey designed specifically for this study it has not been previously validated, and as the current study is an exploratory study validating the survey was not one of its aims.

The beginning of the survey asked participants to report their age (free-text), gender (Male, or Female), role within primary care (free-text), years working in primary care (free-text), and whether they had a special interest or postgraduate qualification in musculoskeletal medicine/surgery (Yes, or No). To meet aim one, the survey asked participants to report whether they recommend self-directed-exercises to patients (Yes, No, or Sometimes), for what conditions they recommend self-directed-exercises (participants could select multiple options, from a list including: back pain, knee pain, tennis elbow, tendinopathy, plantar fasciitis, shoulder pain, hip pain, and other: please specify:[free-text]), and what methods they use to recommend self-directed-exercises (participants could select multiple options, from a list including: explanation only, explanation and demonstration, demonstrations on Youtube, and exercise pamphlets, and could say from where they obtained those resource(s): [free-text]).

Next, to meet aim 2, participants were presented with 28 statements describing barriers and facilitators they might experience when deciding whether to recommend self-directed-exercises. For each statement participants indicated the extent to which they agreed or disagreed on a 7-point Likert scale, where 1 indicated that they strongly agreed and 7 indicated that they strongly disagreed. The survey’s 28 statements were constructed by adapting generic items published in a previous survey based on the Theoretical Domains Framework [32], similar to that done by previous studies that aimed to understand the barriers and facilitators to tobacco cessation and patient safety [33, 34]. The Theoretical Domains Framework includes 14 “domains” that exclusively and exhaustively capture the barriers and facilitators to behavior change [35]. The 14 domains include: Goals, Intentions, Beliefs about consequences, Social/professional role and identity, Behavioral regulation, Emotions, Skills, Reinforcement, Memory, attention, and decision processes, Beliefs about capabilities, Social influences, Knowledge, Environmental context and resources, and Optimism [36].

All 28 statements appear in the first column of Table 2, and the theoretical domains they were designed to capture appear in the final column. Some domains were easily captured by a single statement, while others required multiple statements. For example, the Memory, attention, and decision process domain was captured by a single statement that read: “I feel I should but I don’t remember to do it.” In contrast, the Skills domain was captured by two statements, including: “I feel confident to demonstrate the exercises” and “I feel I can make a persuasive case for exercises.” After responding to each statement, participants were asked to write down actions they thought would increase their tendency to recommend self-directed-exercises. The survey ended with a place for participants to leave their contact information that a researcher could use to invite them to attend a face-to-face interview.

**Table 2:** Theoretical Domains Framework Survey

Statement	Number of Participants	Statement Score		Domain Score		Domain
		Mean	Standard Deviation	Mean	Standard Deviation	
I believe in encouraging patients to self-manage their conditions wherever possible (reverse scored)	108	6.52	0.91	6.32	0.72	Goals
I feel joint exercises are an important part of the management of joint pain and should be encouraged wherever possible (reverse scored)	108	6.13	0.80			
They should only be issued if the patient asks for them	108	6.15	0.78	6.15	0.78	Intentions
A period undertaking exercises allows self-limiting conditions resolve without needing onward referral (reverse scored)	107	6.10	0.81	5.95	0.73	Beliefs about consequences
I believe patients may be harmed by the exercises	104	5.81	1.02			
I feel this is something GPs should be doing (reverse scored)	108	6.25	0.87	5.67	0.87	Social/professional role and identity
This is something I know other GPs do (reverse scored)	107	5.82	1.12			
This is something I have been advised to do (reverse scored)	108	4.94	1.63			
I feel measures like this are an inferior treatment designed to prevent us referring to physiotherapy	108	5.38	1.37	5.38	1.37	Behavioral regulation
I have done this in the past and had negative experiences	107	5.66	1.18	5.34	1.17	Emotions
I am uncertain how patients will react	107	5.00	1.49			
I feel I can make a persuasive case for exercises (reverse scored)	108	5.83	0.92	5.26	0.98	Skills
I feel confident to demonstrate the exercises (reverse scored)	107	4.68	1.57			
Patients really value this kind of advice (reverse scored)	106	5.48	0.96	5.27	0.99	Reinforcement
I feel I am creating more work for myself	108	5.04	1.66			
I feel I should but I don’t remember to do it	107	5.03	1.67	5.03	1.67	Memory Attention and Decision Processes
GPs are not qualified to issue exercises	108	5.56	1.34	4.94	1.15	Belief about capabilities
I do not feel able to provide the follow up the patient requires	108	4.32	1.54			
I feel patients will see this as a way to avoid referring them to physiotherapy	108	4.72	1.48	4.44	1.06	Social Influences
This is something patients ask for (reverse scored)	107	4.13	1.45			
I am not familiar with the practice	106	5.56	1.95	4.43	1.25	Knowledge
I am unsure of the evidence base for self-directed-exercises	106	4.06	1.82			
I don’t know how to construct an exercise programme (e.g. how many times a day for how long)	108	3.65	1.73			
I find it difficult using external resources (e.g. resources from outside the clinical notes / practice intranet) in consultations	108	5.21	1.59	4.38	1.11	Environmental context and resources
The resources (e.g. advice sheets) are easily available (reverse scored)	108	4.87	1.70			
I don’t have the time in my consultations	107	4.40	1.82			
I believe it is important to demonstrate the exercises	107	3.04	1.57			
I don’t believe patents will be compliant with the exercises	106	4.34	1.32	4.34	1.32	Optimism

#### Survey Analyses

To address aim one, participants’ tendency to recommend self-directed-exercises are described using percentages and frequencies. To address aim two, participants Likert scale response to the 28 statements about barriers and facilitators are examined in three ways. First participants’ mean response to each of the 28 statements are described, with responses to statements phrased to indicate facilitators reverse coded so that higher numbers suggest a greater tendency towards recommending self-directed-exercises. Second, where multiple statements were used to capture domains those statements were averaged such that a single number represents each domain as its “domain score.” Third, the domain scores were entered into a multiple regression analyses to ascertain which domains had the greatest influence on participants’ decisions to recommend self-directed-exercises. The findings of the regression analyses are useful in an explorative capacity, but limited in a diagnostic capacity in part due to the small sample-size.

### Interview method

#### Interview Recruitment

A purposive sample of nine survey participants who indicated their willingness to take part in an interview were contacted to be interviewed. These nine participants were chosen to roughly represent the gender and age of general practitioners in the United Kingdom. Rough analyses were conducted after each interview. No new information was found between the eighth and ninth interview. This indicated that data saturation may have been reached by the ninth interview. Data saturation was defined as the point at which no new themes emerged using standard conventions described by Flick [37]. To better ensure data saturation, one additional participant was interviewed and again no new information was found, and so interviewing stopped at 10 participants.

Regarding these 10 participants’ roles in primary care, 9 were general practitioners and 1 was a physiotherapist. The interview results will include all participants. The physiotherapist views are designated as such in the text, and lend an enlightening alternative view point into how the general practitioners interact with adjacent medical professionals. The mean age of the participants was 44 (*Mdn* = 45, *SD* = 9.96), 5 identified as female and 5 identified as male.

#### Interview Materials

A semi-structured interview guide was created to explore areas of interest identified by the survey. For instance, the survey’s results suggested that general practitioners’ tendency to recommend self-directed-exercises was influenced by the evidence supporting self-directed-exercises, and so the second interview question explicitly asked participants “What are your thoughts on the evidence base for self-directed-exercises?” The interview guide questions appears in Table 3. The guide was designed such that interviews would take less than 30-min to conduct. The guide contained eight main questions and probe questions to generate discussion.

**Table 3:** Interview guide with main and probe questions

Question	Main questions
	● Probe questions
1	What are your opinions of SDEs in MSK conditions and problems?
	● Have you any thoughts about GPs issuing SDEs?
	● Is there anything that influences GPs in issuing SDEs?
2	What are your thoughts on the evidence base for SDEs?
	● What is the relevance of the evidence?
	● What is the relevance of the evidence when you are issuing the SDEs?
3	What do patients with MSK conditions think about the provision of SDEs by primary care HCPs?
	● What patient factors are important in this response?
4	What role, if any, do other individuals have to play with SDEs in patients with MSK conditions?
	● What about other healthcare professionals?
	● What about fitness professionals?
	● What about members of the community?
5	What is the role of resources in the provision of self-directed exercises by HCPs?
	● What about printed resources, online resources, etc?
	● What about time?
6	What could be done to encourage patients’ engagement with self-directed exercises?
7	Is there any way that the provision of SDEs by HCPs could be facilitated?
8	Is there anything that you would like to add?

The interviews were conducted by a single researcher, author TG. The interviews took place in a location selected by the participants, including: private residences, general practices, and local Clinical Commissioning Group’s premises. All participants consented to being audio-taped (Sony ICD PX-333). The researcher transcribed the audio-taped interviews verbatim within one-week of the interview with the assistance of InqScribe® [38] following standard conventions, as described by Drew et al. [39].

#### Interview Analyses

The interviews were analyzed by a single researcher, author TG, using an inductive thematic approach, as described by Braun and Clarke [40]. After reading each interview any topic coded at least twice was identified and grouped into sub-themes and superordinate-themes. The themes were then reviewed and refined by reading all the collated extracts for each theme. The transcripts were also re-read to ensure that nothing had been missed and that the initial codes were accurate. The results were discussed with general practitioners to ensure the interpretation of each statement was sound within its context.

## Results

The results section first reviews the results of the surveys and then the interviews.

### Surveys

To address aim one, first the percentage and frequency of participants who reported recommending self-directed-exercises are described. Approximately 73.1% of participants (*N* = 79) indicated recommending self-directed-exercises to patients affected by musculoskeletal problems, 23.1% (*N* = 25) did so sometimes, and only 3.7% (*N* = 4) never did so. Self-directed-exercises were most recommended for back pain (86.1%, *N* = 93), followed by plantar fasciitis (80.6%, *N* = 87), knee pain (75.0%, *N* = 81), tennis elbow (63.9%, *N* = 69), shoulder pain (42.6%, *N* = 46), tendinopathy (54.6%, *N* = 59), and hip pain (63%, *N* = 68). Additionally, 71.3% of participants (*N* = 77) indicated being interested in musculoskeletal medicine.

The largest number of participants recommended self-directed-exercises to patients through exercise pamphlets (77.8%, *N* = 84), followed by explanations and demonstrations (51.9%, *N* = 56), only explanations (21.3%, *N* = 23), and demonstrations via Youtube (19.4%, *N* = 21). The most common source through which participants obtained exercise pamphlets was Arthritis UK (50.9%, *N* = 55), followed by Patient.co.uk (14.8%, *N* = 16), and fewer than 16 participants used any other single source. To increase participants’ tendency to recommend self-directed-exercises, one-third of participants (33.3%, *N* = 36) thought they should have better access to reputable resources, e.g., one participant wrote that a “repository of all accredited exercise demonstration videos” would help, while another thought that “readily accessible leaflets or phone apps” would help. Several participants (11.1%, *N* = 12) thought that they should be given better training, e.g., one participant wrote that general practitioners should have “training on how to demonstrate and advise [exercise] frequency, duration, etc.”

To address the second aim, participants’ responses to the 28 statements about their barriers and facilitators were examined. Table 2 provides each statement in column 1, the number of participants’ responding to each statement in column 2, participants’ mean responses in column 3, and the standard deviations in column 4. Higher scores indicate a greater tendency to recommend self-directed-exercises. Descriptively the statement with the highest score was “I believe in encouraging patients to self-manage their conditions wherever possible” (part of the “Goals” domain, *M* = 6.52, *SD* = 0.91). The statement with the lowest score was “I believe it is important to demonstrate the exercises” (part of the “Environmental context and resources” domain, *M* = 3.04, *SD* = 1.57).

**Fig. 1:**
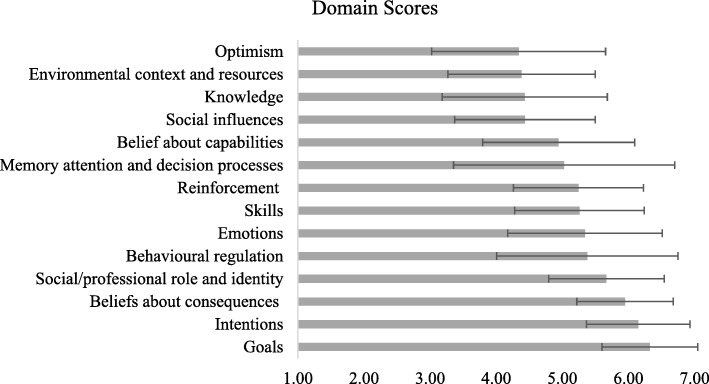
Domain Scores (Error bars represent the standard error of the mean)

Then to yield domain scores, the means of participant’s responses to the statements within each domain were computed. Where participants’ responses were missing, only the available responses were used to compute their mean. In Table 2, the mean domain scores appear in column 5, the standard deviations appear in column 6, and the domain names appear in column 7. Figure 1 displays the mean domain scores from the lowest ranking domain to the highest. The mean domain scores ranged from 4.34 to 6.32. The highest mean domain scores were found for Goals (*M* = 6.32, *SD* = 0.72) and Intentions (*M* = 6.15, *SD* = 0.78), and the lowest were found for Environmental context and resources (*M* = 4.38, *SD* = 1.11) and Optimism (*M* = 4.34, *SD* = 1.32).

Lastly, a multiple linear regression was run to understand which of the 14 domain scores (the independent variables) significantly predicted participants’ tendency to recommend self-directed-exercises (the dependent variable; 0 = No, 1 = Sometimes, 2 = Yes). The results appear in Additional file 2. As a reminder, these findings are useful in an explorative capacity, but limited in a diagnostic capacity in part due to the small sample-size. A significant regression equation was found (*F*(14,101) = 8.70, *p* < .001), with an *R*^*2*^ of .58. Six domains were significant predictors, including Environmental contexts and resources (*β* = .29, *p* = .01), Knowledge (*β* = .29, *p* = .001), Memory attention and decision processes (*β* = .25, *p* = .01), Social/Professional role and identity (β = .22, *p* = .02), Intentions (*β* = −.22, *p* = .02), and Goals (*β* = .19, *p* = .04).

### Interviews

Four themes were identified and are reviewed below. The first theme relates to the Belief in consequences domain, and the second to the Social influences domain. The third and fourth theme relate to the Environmental resources and context domain. The fourth theme is reviewed in the greatest length for two reasons: first, the Environmental resources and context domain theme was identified as a significant predictor in the regression model, and second, this domain likely contains easily malleable factors that can be quickly leveraged to increase general practitioners’ tendency to recommend self-directed-exercises.

#### Theme 1. Positive beliefs about self-directed-exercise influence recommendations

General practitioners were positive about the potential for self-directed-exercises to enhance their patients’ wellbeing. Seven of the nine general practitioners explicitly stated that self-directed-exercises were useful treatment options, at least in the short-term. Seven participants indicated that self-directed-exercises enabled patients to self-manage, and five noted that self-directed-exercises were a very cost-effective. Five of the practitioners clearly stated their confidence that self-directed-exercises were unlikely to harm patients. However, two participants expressed some concerns; One participant cautioned that self-directed-exercises needed to be “taught properly” to benefit patients.

#### Theme 2: Patients’ motivations influence recommendations

The second theme has to do with the general practitioners perceptions of their patients’ motivations to engage in self-directed-exercises. Six participants suggested that patients sometimes appeared to have clear expectations of what treatment should be recommended (e.g., imaging, referrals, or medications), and if self-directed-exercises were not among these expectations, it may be difficult to recommend. One general practitioner suggested that having easy access to a physiotherapist increased patients’ motivations to engage. This participant stated that “patients…come in just wanting to see a physiotherapist for any condition they have,” and reflected that this experience differed from their experience at a previous surgery where access to physiotherapists was not as easy. Two of the participants expressed some caution around the language used with their patients, as terms such as “crumbling” and “exploding” may lead to patients being fearful of doing further damage with exercise.

#### Theme 3: Time available influences recommendations

The third theme has to do with the time limitations of a 10-min general practice consultation. The physiotherapist felt that 10-min was an inadequate length of time to fully assess patients’ conditions and then recommend self-directed-exercises. The physiotherapist compared the 10-min general practitioners often have with the 60-min physiotherapists often have when seeing patients for the first time. The time pressure in general practice was likely a greater barrier when patients presented with multiple conditions, as one participant reported being more likely to recommend self-directed-exercises if patients presented with “a single problem.”

To overcome the time barrier seven practitioners relied on pamphlets. One reported that “I tend to say, it’s all described here and what you need to do. It’s all very self-explanatory.” Three practitioners discussed using the pamphlets to monitor patients’ problems over a number of appointments, e.g., as a workbook or diary. However, while many participants felt the pamphlets were a time-saver, two expressed concerns that their 10-min consultation did not leave sufficient time to help patients understand the information contained in the pamphlets, and therefore the contents of the pamphlets must be made easier to understand (e.g., simple language and diagrams). Note that the desire for pamphlets to save time strongly relates to the fourth theme that will be reviewed now.

#### Theme 4: Making it easy influences recommendations

All of the general practitioners suggested that having better exercise pamphlets would likely increase their tendency to recommend self-directed-exercises. Participants suggested different ways to improve the resources currently available. For example, one participant said that “it would be good if there was some sort of central resource” at which evidenced-based self-directed-exercises could be compiled, along with directions about how to recommend/perform them.

If such a central resource is created, it is likely important that it includes evidence for the efficacy of self-directed-exercises, e.g., academic manuscripts, white literature, and grey literature. One practitioner reflected that “if there is evidence we want to be following it.” Following a direct question about the relevance of the evidence, seven participants expressed uncertainty around the evidence base for self-directed-exercises, and all participants believed that better evidence would make general practitioners more likely to issue recommend them. Two of the general practitioners suggested that the requirement for evidence is less important if an intervention is cheap and unlikely to do harm.

Five of the general practitioners believed that they would be more likely to recommend self-directed-exercises if such recommendations could be more clearly placed in treatment pathways, perhaps combined with a prescription they could hand participants similar to how medications are prescribed. The participants believed that this would not only reinforce the importance of their recommendations to preform self-directed-exercises, but also automatically alert future practitioners who interact with such patients that a recommendation to self-directed-exercises had been made.

Many of the discussions in the interviews covered physiotherapy. Eight of the general practitioners spoke of physiotherapy offering a more comprehensive assessment and treatment of musculoskeletal problems than they were able to provide. One general practitioner spoke very positively about a physiotherapist who worked in his practice. They felt the physiotherapist had a positive effect on the team, by educating general practitioners and improving patient care. Another general practitioner believed that including a physiotherapist in the practice may be cost effective as “you could take out 15% of our workload by employing a physiotherapist.”

## Discussion

To the authors’ knowledge, this is the first study to capture whether general practitioners recommend self-directed-exercises to patients affected by musculoskeletal problems (Aim 1), and to describe the barriers and facilitators they experience to doing so (Aim 2). Regarding aim one, participants’ responses suggest that most (96%) general practitioners recommend self-directed-exercises, at least sometimes. Regarding aim two, the following two domains were the greatest facilitators of general practitioners’ tendency to recommend self-directed-exercises Goals and Intentions, and the following two domains were the greatest barriers to doing so, Environmental contexts and resources and Optimism. The interviews added context to help understand what factors could be leveraged to increase general practitioners’ tendency to recommend self-directed-exercises.

One important finding of the current study (revealed by both the survey and interviews) is that general practitioners recommendations are impeded by time. The limited consultation time, typically 10-min, is a significant problem other studies have noted. For example, one study found that 72% of patients attending a general practice consultations were bothered by multiple problems, with the average patient presenting 2.5 problems [41]. Notably relevant, musculoskeletal problems were the most common. In that study, each additional problem added 2-min to the consultation length, with the average time being 11.9-min, which is nearly 2-min over the time suggested by regulators.

To save time in consultations many participants were already directing patients to use pamphlets and online videos to learn how to do self-directed-exercises. However it should be noted that some participants believed that using the current resources without further explanation would be unwise. The call for better exercise pamphlets was clear from both the surveys and interviews. Indeed, there is a need to produce simpler resources that general practitioners can more quickly explain to patients, as a previous study of elderly patients with osteoarthritis showed that only 10% of patients preformed the exercises correctly after an initial demonstration [42].

Several limitations of the current study should be noted. Three have to do with the validity (external and internal) of the findings. Regarding external validity, the current study’s participants were largely from one region of England, and so it is unknown whether the results will generalize to other areas. Regarding internal validity, the current study’s protocol authorized only one researcher to undertake the transcription and analysis of the interviews. A future study should seek to authorize two researchers to review the data, and then assess inter-rater reliability. Another limitation is that the survey used in the current study is not validated. While some efforts were put forth to ensure the face-validity of the survey, further validating the survey was outside the scope of the current study.

Another limitation is that the survey participants likely over-represent the proportion of general practitioners with a special interest in musculoskeletal issues, i.e., 71.3% participants said that they were interested in it. This over-representation may then produce an overestimate of the degree to which general practitioners already recommend self-directed-exercises, and underestimate some of barriers they experience to do so. One more limitation, is that this work explored the practice of recommending self-directed-exercises to patients presenting with a wide range of musculoskeletal problems, rather than a specific self-directed-exercise for a specific musculoskeletal problem. Further work is required to assess whether general practitioners’ tendencies to recommend some self-directed-exercises differ from others.

Notably, increasing practitioners’ tendency to recommend self-directed-exercises may be insufficient to improve patients’ wellbeing, because the success of self-directed-exercises will depend on patients’ adherence. The need for patients’ adherence here is similar to other treatment recommendations, e.g., diet, medication, etc., though the barriers and facilitators to patients’ adherence likely differ. The current study illuminated general practitioners’ tendency to recommend self-directed-exercises and the barriers and facilitators they experience to doing so. Future research should explore patients’ tendencies to adhere to general practitioners’ recommendations of self-directed-exercises and the barriers and facilitators patients’ experience to doing so.

The current study suggests several things that could be done to increase general practitioners’ tendency to recommend self-directed-exercises to patients affected by musculoskeletal problems. To increase general practitioners’ beliefs in the positive consequences of self-directed-exercises, the current study calls for more rigorous research to evaluate the effectiveness of self-directed-exercises and a central resource to contain these studies. To enhance patients’ motivations to engage in self-directed-exercises, self-directed-exercises should be presented as a clinic supported, easy, and quickly available treatment option. To help general practitioners better manage their consultation time, a reputable source like Patient UK could accredit and make available simple pamphlets about self-directed-exercises for practitioners to give patient [43].

## Conclusions

The current study fulfilled two aims. For aim one, the study revealed that general practitioners largely do recommend self-directed-exercises to patients affected by musculoskeletal problems. For aim two, the study identified several barriers and facilitators general practitioners experience to making such recommendations. Notably, our participants expressed generally positive attitudes towards self-directed-exercises, including that self-directed-exercises likely increase patients’ ability to self-manage, are cost-effective, and are unlikely to cause harm. The current study’s findings can now be used to help general practitioners recommend self-directed-exercises more frequently.

## Additional files

Additional file 1: Survey. (DOCX 16 kb)

Additional file 2: Multiple Regression Table. (DOCX 16 kb)

## Abbreviations

GP: General Practitioner
NHS: National Health Service
NICE: National Institute of Health and Care Excellence
