# Supplementary material for: General Practitioners' recommendations of self-directed-exercises for musculoskeletal problems and perceived barriers and facilitators to doing so: a mixed methods study
Source: BMC Health Serv Res. 2018 Dec 27;18:998. doi: 10.1186/s12913-018-3799-x (PMC6307153; doi:10.1186/s12913-018-3799-x)
Supplement: Supplementary file 1 — Survey. (DOCX 16 kb) [file 12913_2018_3799_MOESM1_ESM.docx]

**Additional File 1**

**Survey**

Thank you for taking the time to complete this questionnaire.

It aims to explore whether clinicians advise self-directed exercise to their patients with musculoskeletal conditions, and identify which factors, if any, influence clinicians in their decision to advise self-directed exercise.

This questionnaire is entirely anonymous. However, if you would be happy to discuss your answers further, please provide your contact details, including email and telephone number, at the bottom of this questionnaire.

Thank you again.

Please circle the appropriate answer

1. Are you male or female?

- Female
- Male

1. What is your age? _____
2. What is your position

- GP
- GP registrar
- Minor illness practitioner/ nurse practitioner
- Other (please specify)

1. Which Clinical Commissioning Group (CCG) do you mainly practice in?

- Aylesbury Vale CCG
- Chiltern CCG
- Oxfordshire CCG
- Bedfordshire CCG
- Milton Keynes CCG
- Other (please specify)

1. How many years have you worked in primary care (approximately)?

_____

1. Do you have a special interest or postgraduate qualification in musculoskeletal medicine/surgery?

- Yes
- No

1. Do you issue self-directed exercises to patients presenting with musculoskeletal conditions (please circle)?

- Yes
- No
- Sometimes

1. If you do issue exercises can you indicate which conditions you do it for:

- Back pain
- Knee pain
- Tennis elbow
- Achilles tendinopathy
- Plantar fasciitis
- Shoulder pain
- Hip pain
- Other (please specify)

__________________________________________________________________________________________________________________________________

1. If you do advise self-directed exercises, which method(s) do you use?

- Explanation only
- Explanation and demonstration
- Demonstrations on Youtube
- Exercise pamphlets – please indicate which resource(s) you use e.g. Arthritis Research UK

_________________________________________________________________

_________________________________________________________________

_________________________________________________________________

1. What are your opinions about GPs issuing self-directed-exercises? Please circle the number next to each statement that best reflects your opinion.

| Statement | Strongly Agree to Strongly Disagree |
| --- | --- |
| I am not familiar with the practice | 1 2 3 4 5 6 7 |
| This is something I know other GPs do | 1 2 3 4 5 6 7 |
| I feel this is something GPs should be doing | 1 2 3 4 5 6 7 |
| This is something I have been advised to do | 1 2 3 4 5 6 7 |
| GPs are not qualified to issue exercises | 1 2 3 4 5 6 7 |
| They should only be issued if the patient asks for them | 1 2 3 4 5 6 7 |
| I am unsure of the evidence base for self-directed-exercises | 1 2 3 4 5 6 7 |
| I don’t know how to construct an exercise programme (e.g. how many times a day for how long) | 1 2 3 4 5 6 7 |
| I feel confident to demonstrate the exercises | 1 2 3 4 5 6 7 |
| I believe it is important to demonstrate the exercises | 1 2 3 4 5 6 7 |
| I feel I can make a persuasive case for exercises | 1 2 3 4 5 6 7 |
| I don’t believe patents will be compliant with the exercises | 1 2 3 4 5 6 7 |
| I believe patients may be harmed by the exercises | 1 2 3 4 5 6 7 |
| A period undertaking exercises allows self-limiting conditions to resolve without needing onward referral | 1 2 3 4 5 6 7 |
| I feel joint exercises are an important part of the management of joint pain and should be encouraged wherever possible | 1 2 3 4 5 6 7 |
| I feel I am creating more work for myself | 1 2 3 4 5 6 7 |
| This is something patients ask for | 1 2 3 4 5 6 7 |
| I feel I should but I don’t remember to do it | 1 2 3 4 5 6 7 |
| I don’t have the time in my consultations | 1 2 3 4 5 6 7 |
| The resources (e.g. advice sheets) are easily available | 1 2 3 4 5 6 7 |
| I find it difficult using external resources (e.g. resources from outside the clinical notes / practice intranet) in consultations | 1 2 3 4 5 6 7 |
| I am uncertain how patients will react | 1 2 3 4 5 6 7 |
| I feel patients will see this as a way to avoid referring them to physiotherapy | 1 2 3 4 5 6 7 |
| I feel measures like this are an inferior treatment designed to prevent us referring to physiotherapy | 1 2 3 4 5 6 7 |
| Patients really value this kind of advice | 1 2 3 4 5 6 7 |
| I do not feel able to provide the follow up the patient requires | 1 2 3 4 5 6 7 |
| I have done this in the past and had negative experiences | 1 2 3 4 5 6 7 |
| I believe in encouraging patients to self-manage their conditions wherever possible | 1 2 3 4 5 6 7 |

Other (please specify)

1. Are there any measures you can think of that would make you more likely to suggest self-directed exercises?

_________________________________________________________________

_________________________________________________________________

_________________________________________________________________

1. If you are happy to be contacted to discuss your answers further please leave your details below:

- Name
- Email address
- Phone number
