# Supplementary material for: General Practitioners' recommendations of self-directed-exercises for musculoskeletal problems and perceived barriers and facilitators to doing so: a mixed methods study
Source: BMC Health Serv Res. 2018 Dec 27;18:998. doi: 10.1186/s12913-018-3799-x (PMC6307153; doi:10.1186/s12913-018-3799-x)
Supplement: Supplementary file 2 — Multiple Regression Table. (DOCX 16 kb) [file 12913_2018_3799_MOESM2_ESM.docx]

**Additional File 2**

**Multiple Regression Table**

*Summary of Regression Analysis for Variables Predicting tendency to recommend self-directed-exercises (N = 108)*

|  | *Unstandardized Coefficients* | | | *Standardized Coefficients* | | |  | |  |
| --- | --- | --- | --- | --- | --- | --- | --- | --- | --- |
| Variable | *B* | | *Standard error* | | *B* | | *p* | |  |
| (Constant) | -0.594 | |  | |  | | 0.192 | |  |
| Environment | 0.139 | | 0.048 | | 0.293 | | 0.005** | |  |
| Knowledge | 0.123 | | 0.037 | | 0.291 | | 0.001** | |  |
| Memory Attention & Decision | 0.080 | | 0.028 | | 0.249 | | 0.006** | |  |
| Social/Professional Identity | 0.134 | | 0.054 | | 0.220 | | 0.015* | |  |
| Intention | -0.149 | | 0.061 | | -0.219 | | 0.016* | |  |
| Goals | 0.141 | | 0.069 | | 0.188 | | 0.044* | |  |
| Skills | 0.095 | | 0.050 | | 0.175 | | 0.062 | |  |
| Emotions | -0.078 | | 0.042 | | -0.166 | | 0.068 | |  |
| Reinforce | 0.086 | | 0.086 | | 0.157 | | 0.128 | |  |
| Behavioral Regulation | -0.039 | | 0.034 | | -0.100 | | 0.259 | |  |
| Belief in Capabilities | -0.024 | | 0.043 | | -0.051 | | 0.577 | |  |
| Optimism | -0.014 | | 0.035 | | -0.035 | | 0.688 | |  |
| Social Influences | -0.014 | | 0.040 | | -0.027 | | 0.740 | |  |
| Belief in Consequences | -0.013 | | 0.065 | | -0.017 | | 0.847 | |  |
| *R^2^* | 0.58 | |  | |  | |  | |  |
| *F* for change in *R^2^* | 8.70 | |  | |  | |  | |  |
| **p* < .05. ***p* < .01. |  |  | | | |  | |  | |
